# Continuously superior-strong carbon nanofibers by additive nanostructuring and carbonization of polyacrylonitrile jetting

**DOI:** 10.1038/s41378-024-00800-7

**Published:** 2024-12-10

**Authors:** Jufeng Deng, Chong Liu, Marc Madou

**Affiliations:** 1 Key Laboratory of Advanced Manufacturing Technology of the Ministry of Education, Guizhou University, Guizhou, 550025 China; 2 School of Mechanical Engineering, Dalian University of Technology, 116023 Dalian, China; 3 Mechanical and Aerospace Engineering, University of California, Irvine, CA 92617 USA; 4 School of Engineering and Science, Tecnologico de Monterrey, Ciudad de México, 64849 Mexico

**Keywords:** Electrical and electronic engineering, NEMS

## Abstract

Carbon nanofibers show the advantages of scale effects on electrical and mechanical properties for applications such as aerospace^1,2^, automotive^3,4^, and energy^5,6^, but have to confront the challenge of maximizing the role of scale effects. Here, a method of additive nanostructuring and carbonization of polyacrylonitrile (PAN) jetting for the nano-forming of carbon fibers is developed by understanding the electrostatic submicro-initiation of a PAN jetting, altering the microstructure of PAN-based jetting fibers at the nanoscale and implementing subsequent carbonization of PAN jetting nanofiber. Using this method of additive nanostructuring and carbonization in combination with the radial distribution pattern of shear stress, we find that the conformation of some molecular chains inside the PAN nanofibers is transformed into the zigzag conformation. The ability to materialize and carbonize such PAN nanofibers with various conformational structures in the form of arrays on diverse micro-structures and macro-substrates enables the forming of continuous carbon nanofibers with a diameter of ~20 nm and allows the tensile strength of carbon fibers to be enhanced to 212 GPa through the combination of zigzag conformation and nanoscale effects. These advantages create opportunities for the application of maximizing nanoscale effects that have not previously been technically possible.

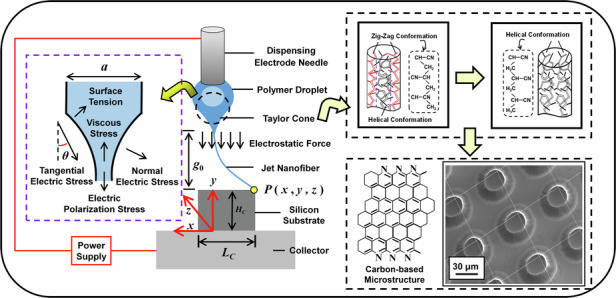

## Introduction

An attractive feature of polyacrylonitrile (PAN) jetting derives from the effect of jetting continuity, microscopic structure, and scale on the high performance of carbon-nanowires-based micro/nano-structure for energy storage devices^7–10^, such as electrochemical energy storage, lithium-ion batteries, and supercapacitors. The effect is expected to be highlighted by adjusting structural conformation at the microscopic level for crystalline microscopic structure. The adjustment is made possible to orient these CN groups within the PAN fibers and transform microstructural conformation from a 3^1^helix to a zigzag configuration by the polarization and stretching process^11^. The transformation of microstructure conformation allows the generation of new PAN materials with excellent hydrophilicity, mechanical and thermal properties, and piezoelectric properties that are compared with those of PVDF^11^. These excellent properties are anticipated to be embodied in macroscopic carbon-fiber-based chips and devices by carbonizing such PAN fiber into continuous carbon nanofibers at the nanoscale for highly specific surface area and superior properties. Therefore, additive nanostructuring and carbonization of PAN jetting become essential for continuously superior-strong carbon nanofibers.

To achieve this additive nanostructuring and carbonization of PAN jetting, specific aging procedures of the PAN/N, N-dimethyl formamide solution, and sequential special electrospinning and post-thermal treatment procedures have been employed to pole and stretch the molecular chains in the PAN fibers^12,13^. The resulting molecular chains show a greater fraction of the zigzag confirmation (an electroactive phase) over the helical (a nonelectroactive phase) conformation but are accompanied by some defects such as the beading and clumping at the microscopic level and a disorderly arrangement of PAN nanofibers at macroscopic level. These specified defects can be overcome with the use of low-voltage near-field electrospinning (NFES)^14^, but the raw material for NFES is polyethylene oxide and not PAN. Upon selecting PAN as a raw material, the resolution for the forming of this fiber has increased from 16.2 nm to ~290 nm due to the increase in the working voltage from 200 to 400 V^15^. Given an increasing trend of average diameter versus working voltage^14,15^ and the effect of an extender cap on working voltage^16^, the resolution of PAN fiber can be improved to near-nanometer scale using the touch and retract mode of the droplet at the ejector needle tip and a rotating drum in NFES^17–19^. Although this touch and retract mode offers a possibility to evolve NFES into an additive nano-manufacturing, the additive nanostructuring and carbonization of PAN jetting have to be challenged in realizing well-organized nano-sized diameter at nano-forming level, featuring orientation of the dipole groups and polymer chains at the nano-structuring level, and materializing such PAN fibers on micron/macro-structures and their composite structures at the nano-additive level.

In this work, the nano-forming of PAN jetting for the well-organized nano-sized diameter is theorized by establishing and analyzing a mathematical model involving electric stress on the droplet meniscus, shear stress of the fluid, and minimum steady-state voltage in the electrostatic jetting. Using these theories, the orientation of PAN molecular chains and the materialization of such PAN nanofibers onto various structures are available by designing and experimentally implementing the nano-forming and nano-structuring processes of PAN jetting fiber. The subsequent carbonization of these PAN nanofibers with the orientation of molecular chains is executed for continuous carbon nanofibers, and the mechanical properties of resulting carbon nanofibers are measured by atomic force microscopy (AFM) using the stretching of AFM tip.

## Mathematical modeling and theoretical analysis

### Electric stress on the meniscus of PAN droplet

**Fig. 1:**
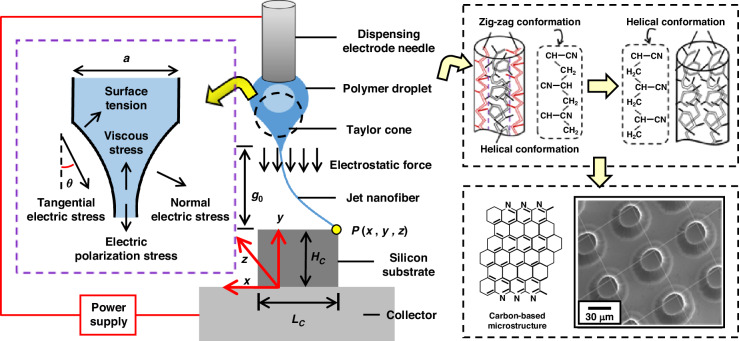
Schematic of forming continuously superior-strong carbon nanofibers by additive nanostructuring and carbonization of polyacrylonitrile jetting

As illustrated in Fig. 1, continuously superior-strong carbon nanofibers are formed by additive nanostructuring and carbonization of polyacrylonitrile (PAN) fiber jetting, which are accompanied by the formation of zigzag conformation and carbon-based microstructure as well as the transition from zigzag conformation to helical conformation. The process of electrostatic PAN-fiber jetting is initiated by the adsorption of a liquid droplet onto the surface of the collector and the induction of an artificial instability at the droplet–air interface^19^. The adsorption and the artificial instability are implemented by applying a voltage over a very small gap between the droplet and the collector, thereby generating a very high local electric stress. The reduction of this voltage allows the diameter of the electrostatic PAN jetting to be decreased to submicron^11^. In the case of the further reduction of the diameter in PAN jetting from submicron to nanometer, the following mathematical modeling of electric stress based on a lower voltage is implemented to obtain a control method of electric stress for PAN-based nano-jetting.

Given the nature of the PAN solution^11^ in a small droplet at the needle tip, a Newtonian fluid is assumed, and the governing equations for the adsorption of that droplet onto the collector surface are described as follows:

1 $$\left\{\begin{array}{l}{F}_{ele}-{\tau }_{\mu }=m\frac{{d}^{2}y}{d{t}^{2}}\\ {F}_{ele}=\frac{\varepsilon A{V}^{2}}{2{({g}_{0}-y)}^{2}},{\tau }_{\mu }=\frac{\mu }{a}y\end{array}\right.$$

where *F*_ele_ is an electrostatic force on the droplet at the tip of the dispensing electrode needle as shown in Fig. 1, *τ*_μ_ is viscous stress in the tangential direction, *m* and *y* are the mass and the axial displacement, respectively, *Ɛ* is the dielectric constant of the liquid, *A* is the area of the fluid that faces the collector, *V* is the applied voltage between the dispensing electrode needle and the collector, *g*_0_ is the axial distance between the fluid and the collector, *μ* is an absolute viscosity of the liquid, *a* is the width of the fluid at the tip of the dispensing electrode needle.

For the critical case of the adsorption between the liquid droplet and the collector surface, the following conditions need to be satisfied:

2 $$\begin{array}{l}\left\{\begin{array}{l}\frac{{d}^{2}y}{d{t}^{2}}=0\\ \displaystyle{\int_{0}^{{Y}_{1}}}\,\frac{\varepsilon A{V}^{2}}{2{({g}_{0}-y)}^{2}}dy-{\int_{0}^{{y}_{1}}}\frac{\mu }{a}ydy={\int_{{y}_{1}}^{{y}_{2}}}\,\frac{\mu }{a}ydy-{\int_{{y}_{1}}^{{y}_{2}}}\,\frac{\varepsilon A{V}^{2}}{2{({g}_{0}-y)}^{2}}dy\,(V={V}_{R})\end{array}\right.\end{array}$$

where *y*_1_ and *y*_2_ are displacements of the liquid on the surface of the droplet when the electrostatic force is equal to the viscous stress, and *V*_R_ is the threshold voltage for droplet adsorption.

By combining Eq. 2 and Eq. 1, the final expressions for this threshold voltage are derived as follows:

3 $$\left\{\begin{array}{l}\,{V}_{R}=\sqrt{\frac{{g}_{0}\mu {y}_{2}({g}_{0}-{y}_{2})}{\varepsilon Aa}},\,{y}_{2}=\frac{2{g}_{0}}{3}\,(1+\,\cos \frac{\theta +\pi }{3})\\ \quad\theta =\arccos (1-\frac{108\varepsilon aA{{V}_{R}}^{2}}{16\mu {{g}_{0}}^{3}})\end{array}\right.$$

In terms of adsorption at the threshold voltage, the electric stress on the surface of the liquid droplet *τ*_*E*_ can be given as follows:

4 $${\tau }_{E}=\sigma {E}_{t}c{\rm{os}}{\theta }_{1}=\frac{\sigma {V}_{R}(\cos 2{\theta }_{1}+1)}{2({g}_{0}-{y}_{2})}$$

where *σ* is the surface charge, *E*_*t*_ is the tangential electric field strength at the liquid surface, *θ*_1_ is the angle between the tangential electric stress and the *y*-axis (see Fig. 1).

Substituting Eq. 3 into Eq. 4, the electric stress *τ*_*E*_ is obtained as follows:

5 $$\left\{\begin{array}{l}{\tau }_{E}=\sigma \,(\cos 2{\theta }_{1}+1)\sqrt{\frac{\mu {y}_{1}}{4a\varepsilon A(1-\frac{{y}_{2}}{{g}_{0}})}}\\ {y}_{2}=\frac{2{g}_{0}}{3}(1+\,\cos \frac{\theta +\pi }{3}),\,\theta =\arccos [1-\frac{108{y}_{1}({g}_{0}-{y}_{2})}{16{{g}_{0}}^{2}}]\end{array}\right.$$

Investigation into the effects of the factors in Eq. 5 on the electric stress of droplets reveals a decrease in the electric stress versus the axial distance between the fluid and the collector (g_0_). The electric stress is at a maximum when the axial distance is near zero, inducing instability at the droplet–air interface. This explains well that the reduction of the axial distance to near zero can induce artificial instability at the droplet–air interface due to the maximation of the electric stress^19^, enabling the thicknesses of polymer fibers as low as near the nanometer scale. Moreover, Eq. 5 shows that a decrease in the viscosity of the liquid with a lower axial distance allows the electric stress to be further increased, making a lower threshold voltage for electrostatic jetting^17^. Combined with a lower axial distance between the fluid and the collector and a smaller liquid viscosity, a decrease in the fluid width at the tip of the dispensing electrode needle enables a further maximization in the electric stress in Eq. 5 to initiate the electrostatic jetting at yet lower threshold voltages.

### Shear stress on the fluid inside the dispensing electrode needle

During electrostatic jetting, the liquid inside the dispensing electrode needle is assumed to be isothermal, incompressible, and laminar. With a steady flow of the liquid fully developed, the pressure *p* and stress tensor *τ* are related as^20^:

6 $$-\nabla p+\nabla \cdot \tau =0$$

In the case of a straight circular needle, the shear stress along the flow direction perpendicular to the radial direction in Eq. 6 is described as:

7 $${\tau }_{\mu x}=-\frac{1}{2}\frac{dp}{dy}x=\frac{1}{2}\frac{\varDelta p}{l}x$$

where Δ*p* is the pressure difference between the outlet and inlet of the dispensing electrode needle, and *l* is the length of the dispensing electrode needle.

From Eq. 7, the viscous shear stresses located from the center of the needle axis to the inner wall of the needle are linearly distributed along the needle radius direction. On the axis of the dispensing electrode needle, the shear stress is zero, and on the inner wall surfaces, the shear stress is maximum. Importantly those shear stresses on the liquid allow the PAN molecular chains in the liquid to be nano-structured in accordance with the characteristics of the shear stress distribution: these PAN molecular chains line up along the needle walls while remaining chaotic in the center. This provides a theoretical basis for the conformational change of PAN molecular chains, which well bridges the transition from the nano-forming of PAN jetting fiber^19^ to the nanostructuring of PAN molecular chains.

### Minimum steady-state voltage for electrostatic jetting

Upon initiating electrostatic jetting in NFES, a steady-state voltage is used to maintain sufficient electric stress for the continuity of the jet, thus avoiding interruption of the liquid bridge between the dispensing electrode needle and the collector. With an axisymmetric liquid cone in steady-state, the voltage allows the change in potential energy (pressure *p*_liq_ and gravitation *p*_g_) and kinetic energy *p*_Ekin_ (velocity pressure) to be balanced with the energy input due to the tangential electric stress *τ*_Ey_, the change in polarization stress *σ*_ε_ and the energy dissipation due to the viscous stress in the liquid. This force balance in the *y* direction of the cone (see Fig. 1), from the Navier-Stokes equation, is described as^21^:

8 $$\displaystyle\left\{\begin{array}{l}\begin{array}{ccc}\displaystyle\frac{\partial ({p}_{Ekin}+{p}_{liq}-{\sigma }_{\mu }-{\sigma }_{\varepsilon })}{\partial y}=\frac{2}{{x}_{s}}({\tau }_{\mu }+{\tau }_{Ey}),{p}_{Ekin}=\frac{1}{2}{C}_{profile}\rho {(\overline{{u}_{y}})}^{2}\end{array}\\ {p}_{liq}={p}_{out}+\mu \frac{2{(d{x}_{s}/dy)}^{2}-1}{{({\rm{d}}{x}_{s}/dy)}^{2}+1}\times \frac{\partial {u}_{y}}{\partial y}+\gamma (\frac{1}{{x}_{s1}}+\frac{1}{{x}_{s2}})\\ \begin{array}{cc}\begin{array}{ccc}\qquad\,\,\displaystyle-\frac{1}{2}{\varepsilon }_{0}({E}_{n,out}^{2}-2{\varepsilon }_{r}{E}_{n,ins}^{2}+{E}_{n,ins}^{2})\end{array}\end{array}\\ \begin{array}{ccc}\displaystyle{\sigma }_{\mu }=2\mu \frac{\partial {u}_{y}}{\partial y},\,{\sigma }_{\varepsilon }=\frac{1}{2}{\varepsilon }_{0}({\varepsilon }_{{\rm{r}}}-1)({E}_{n,ins}^{2}+{E}_{t}^{2})\end{array}\\ \begin{array}{ccc}\displaystyle{\tau }_{\mu }=\frac{3\mu (\partial {{\rm{u}}}_{y}/\partial y)(d{x}_{s}/dy)}{1+{(d{x}_{s}/dy)}^{2}},\,{\overline{u}}_{y}=\frac{Q}{\pi {x}_{s}^{2}}\end{array}\end{array}\right.$$

where *C*_profile_ is a correction factor for the radial profile of the axial velocity distribution inside the liquid cone, *ρ* is the fluid density, *u*_y_ is the liquid velocity in the axial direction, *x*_s_ is the radius at the surface of the liquid cone, *γ* is the surface tension, *x*_s1_ and *x*_s2_ are radii of curvature at the surface, *p*_out_ is the air pressure, *ε*_0_ is a vacuum permittivity, *ε*_r_ is a relative permittivity of the liquid, *E*_n,out_ is the electric field strength normal to the liquid-air surface outside the liquid, *E*_n,ins_ is the electric field strength normal to the liquid-air surface inside the liquid, *σ*_μ_ and *τ*_μ_ are the normal and tangential viscous stresses respectively.

Since the force *F*_k_ needed to accelerate the fluid toward the cone tip is nondimensionalized with the liquid area at the nozzle exit *A*_N_ and the jet cross-sectional area *A*_j_, Eq. 8 is transformed into Eq. 9 as below:

9 $$\left\{\begin{array}{l}\frac{\partial ({p}_{Ekin}+{p}_{liq}-{\sigma }_{\mu }-{\sigma }_{\varepsilon })}{\partial y}=\frac{2}{{x}_{s}}({\tau }_{\mu }+{\tau }_{Ey}),{\tau }_{Ey}=\sigma {E}_{y},({E}_{y} \sim {V}_{\min })\\ {p}_{Ekin}=\frac{{F}_{{\rm{k}}}}{{A}_{N}}\approx [\frac{1}{2}\rho {Q}^{2}(\frac{1}{{A}_{j}}-\frac{1}{{A}_{N}})]/{A}_{N}=\frac{1}{2}\rho {Q}^{2}(\frac{1}{{A}_{j}{A}_{N}}-\frac{1}{{A}_{N}^{2}})\end{array}\right.$$

where *E*_*y*_ is the tangential electric field strength in the axial direction, *V*_min_ is a minimum steady- state voltage for the onset of the cone-jetting, *Q* is a volume flow rate.

From Eq. 9, a decrease in the liquid area at the nozzle exit (*A*_*N*_) facilitates the reduction of the minimum steady-state voltage for continuous electrostatic jetting. Taking the relationship between the liquid area at the nozzle exit and the total volume in the liquid cone into consideration, the minimum steady-state voltage will decrease as the volume of liquid in the cone decreases. In addition, previous studies have shown^17–19^ that a reduction in the minimum value of the steady-state voltage is crucial for decreasing the diameter of the jetting fiber. Consequently, the nano-forming of PAN jetting fibers depends sensitively on the decrease in the volume of the liquid cone at the tip of the dispensing electrode needle.

### Diameter of PAN-based fibers in electrostatic jetting

In electrostatic jetting at the minimum steady-state voltage, the liquid jet moves towards the collector and is converted into fibers with the volatilization of a portion of the solvent. Much volatilization is not allowed due to lower volatility in the short spacing between the dispensing electrode needle and the collector, so the diameter of the jet-based fiber can be considered to be equal to that of the liquid jet. In order to obtain a mathematical model for the diameter of the jet-based fiber, the governing equations for the current *I* and the jetting velocity *v* in steady-state flow at the minimum steady-state voltage are combined with the conservation of charge. In this case, these equations are converted into these formulas as follows:

10 $$\left\{\begin{array}{l}\displaystyle\sigma v(d/2)+\frac{{K}^{\ast }}{2}{(d/2)}^{2}{E}_{y}=I\begin{array}{cc}, v=\frac{Q}{{(d/2)}^{2}}\end{array}\\ \begin{array}{ccc}\displaystyle{K}^{\ast }=K{[{r}_{0}^{3}\rho /(\gamma \beta )]}^{1/2},\,\beta ={\varepsilon }^{3/2}-1\end{array}\end{array}\right.$$

where *d* is the cross-sectional diameter of the jet, *K* is the electrical conductivity of the jet fluid, *r*_0_ is a length scale determined by the nozzle diameter^22^.

In continuous jetting mode, the tangential electric stress becomes the main force and is balanced by the inertial force. The resulting Navier-Stokes equation consists of these two terms only and then is evolved into this formula as follows:

11 $$\frac{d}{dy}(\frac{{v}^{2}}{2})=\frac{2\sigma {E}_{y}}{(\frac{d}{2})\sqrt{\beta }}$$

By combining Eq. 10 with Eq. 11, an expression for the diameter of the jet-based fiber is obtained as follows:

12 $$\begin{array}{c}d=2InverseFunction\left\{\left[-\frac{{{E}_{y}}^{2}{K}^{2}Iny}{8{I}^{3}}+\frac{{{E}_{y}}^{2}{K}^{2}In(-2I+{E}_{y}K{y}^{2})}{16{I}^{3}}\right.\right.\\\left.\left. +\frac{1}{8I{y}^{4}}+\frac{{E}_{y}K}{8{I}^{2}{y}^{2}}\right]\,\left[\frac{{E}_{y}y}{2{Q}^{3}\sqrt{\beta }}+{C}_{1}\right]\right\}({E}_{y} \sim {V}_{\min })\end{array}$$

where *C*_1_ is a constant.

As the tangential electric field strength in the axial direction tends to zero in the case where the minimum steady-state voltage gradually decreases to near zero, Eq. 10 is transformed into *Qσ*/(*d*/2) = *I*. By further combining *Qσ*/(*d*/2) = *I* with Eq. 12, the diameter of the jetting fiber in this case is obtained as follows:

13 $$d=2{\left(\frac{4I{E}_{y}}{\sqrt{\beta }{Q}^{3}}\right)}^{-1/4}{y}^{-1/4}\begin{array}{cc} & ({E}_{y} \sim {V}_{\min }\to 0)\end{array}$$

As mentioned above, the final expressions for the diameter of the jetting fiber are given as follows:

14 $$d=\left\{\begin{array}{l}=2{InverseFunction}\left\{\left[-\frac{{{E}_{y}}^{2}{K}^{2}Iny}{8{I}^{3}}+\frac{{{E}_{y}}^{2}{K}^{2}In(-2I+{E}_{y}K{y}^{2})}{16{I}^{3}}\right.\right.\\ \left.\left.\quad+\,\frac{1}{8I{y}^{4}}+\frac{{E}_{y}K}{8{I}^{2}{y}^{2}}\right]\right\}\left[\frac{{E}_{y}y}{2{Q}^{3}\sqrt{\beta }}+{C}_{1}\right]({E}_{y} \sim {V}_{\min }={\rm{constant}})\\ =2{\left(\frac{4I{E}_{y}}{\sqrt{\beta }{Q}^{3}}\right)}^{-1/4}{y}^{-1/4}({E}_{y} \sim {V}_{\min }\to 0)\end{array}\right.$$

From Eq. 14 the tangential electric field strength in the axial direction is proportional to the minimum steady-state voltage, and the decrease in the diameter of the jetting fiber depends on the reduction of the tangential electric field strength. This demonstrates the strong dependence of the jetting fiber diameter on the minimum steady-state voltage. By reducing the minimum steady-state voltage as much as possible, the diameter of the jetting fiber can be brought down (at *V*_min_ = constant in Eq. 14. In case of minimum steady-state voltage, a subsequent increase in the spacing between the dispensing electrode needle and the collector (see Eq. 14) further facilitates the decrease in the diameter of the jetting fiber. Summarizing, the combination of a decrease in minimum steady-state voltage and an increase in the spacing between the dispensing electrode needle and the collector (in the case of *E*_y_ ~ *V*_min_ = constant) enables an effective decrease in the diameter of jetting fiber for the nano-forming and subsequent nanostructuring of PAN jetting fibers. Furthermore, Eq. (14) shows the first decreasing and then increasing trend of the fiber diameter with the increase of the minimum steady-state voltage, which clearly compensates for previous studies regarding the decreasing tendency of the fiber diameter with increasing voltage^19^.

## Results and discussion

### Nano-structuring of molecular chains for the submicro-forming of PAN-based fiber

**Fig. 2:**
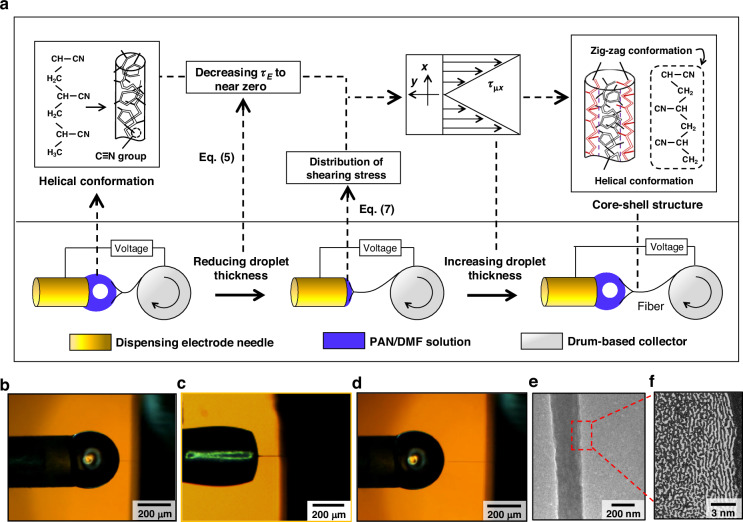
. **Nano-structuring of molecular chains for PAN-based submicron-fiber at 500**  **V** **a** Schematic design of the nano-structuring process based on the submicron-forming of PAN-based fibers. **b**–**d** Micro-manipulation of jetting-based droplet during continuous submicron-jetting by reducing the droplet volume to near zero in **b**, **c**, and then increasing the volume in **c**, **d**. **e** A transmission electron microscopy image of the PAN jetting fiber. **f** A magnification of the pattern in **e**

During the submicro-forming for PAN jetting fiber, the nano-structuring of molecular chains is implemented by the use of the radial distribution pattern in the shear stress (see Eq. 7). A prerequisite for this radial distribution pattern in the shear stress to be maintained is the pulling away of the droplet at the tip of the dispensing electrode needle. By pulling away the droplet at the tip of the dispensing electrode needle, this radial distribution pattern of the shear stress can act on the molecular chains of jet. Taking this case into account, the process of nano-structuring in the molecular chains is designed based on the jetting process and the subsequent reduction in droplet thickness, as shown in Fig. 2a.

In accordance with this design, the nano-structuring process of PAN-based fibers in Fig. 2b–d is achieved by initiating the jetting in Fig. S1, decreasing the flow rate of the fluid, bringing the rotating drum to shear off the droplets with the decrease in the needle-drum, and carrying away a part of the liquid. This allows the molecular chains to be subjected to a radial shear distribution pattern without the interruption of the jetting from the meniscus of the droplet. From characterization with transmission electron microscope in Fig. 2e–f, the resulting molecular chains on the inner wall of the needle at the maximum shear stress show a zigzag conformation. In contrast, the molecular chains in the center of the needle at the low shear stress show a helical conformation. The core-shell structure in PAN-based submicro-fiber can be formed by the zigzag conformation and the helical conformation. These results indicate that the process of nano-structing in the molecular chains can is capable of organizing molecular chains into core-shell structures for PAN-based submicro-fiber.

### Nano-structuring control for molecular chains in PAN-based fiber

**Fig. 3:**
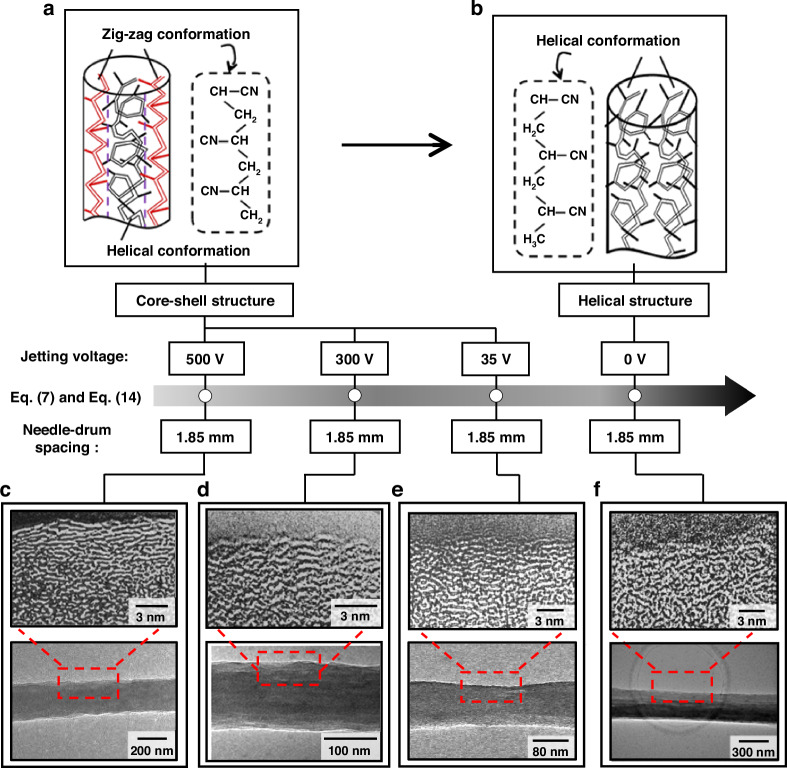
**Nano-structuring of molecular chains for the transition from submicron to nanoscale in the forming of PAN jetting fiber** **a**, **b** Schematic nano-structuring of molecular chains with the helical and zigzag conformations for a transition from helical structure (**a**) to core-shell structure (**b**). **c**–**f** High-resolution transmission electron microscopy images of PAN-based jetting fibers at the needle-drum spacings of 1.85 mm with the minimum steady-state voltages of 500 V (**c**), 300 V (**d**), 35 V (**e**), and 0 V (**f**), respectively

In the submicron-to-nanoscale forming of PAN jetting fiber (Figs. S2 and S3), nano-structuring control for molecular chains can be feasible based on the radial shear stress distribution in the dispensing needle. The stretching effect of maximum shear stress in this distribution on the molecular chains shows the ability to form the zigzag conformation at 500 V, as shown in Fig. 2e, f. By reducing the minimum steady-state voltage from 500 V to 0 V, the maximum shear stress decreases down to zero. In this case, this stretching effect becomes so weak that the shear stress hardly acts on the molecular chain, thus obtaining a helical structure. This demonstrates that the nano-structuring of molecular chains in PAN-based fiber can be manipulated by the control of the minimum steady-state voltage, as shown in Fig. 3a, b.

In Fig. 3c–f, we show HRTEM images of PAN-based fibers at a needle-drum spacings of 1.85 mm and at the minimum steady-state voltages of 500 V, 300 V, 35 V, and 0 V, respectively. In the range from 500 V to 35 V, a core-shell structure with a zigzag configuration on the outside and helical on the inside is shown in Fig. 3c–e. At 0 V, a helical structure dominated by helical configuration is presented in Fig. 3f. By reducing the minimum steady-state voltage from 500 V to 0 V, the transition from the core-shell structure to the helical structure is achieved, showing the ability to control the nano-structuring of molecular chains in PAN-based fiber. This indicates that nano-structuring control for molecular chains can contribute to the forming of core-shell structures in PAN-based nanofiber.

### Additive of PAN jetting fibers with core-shell structure for micro/nano-structure

**Fig. 4:**
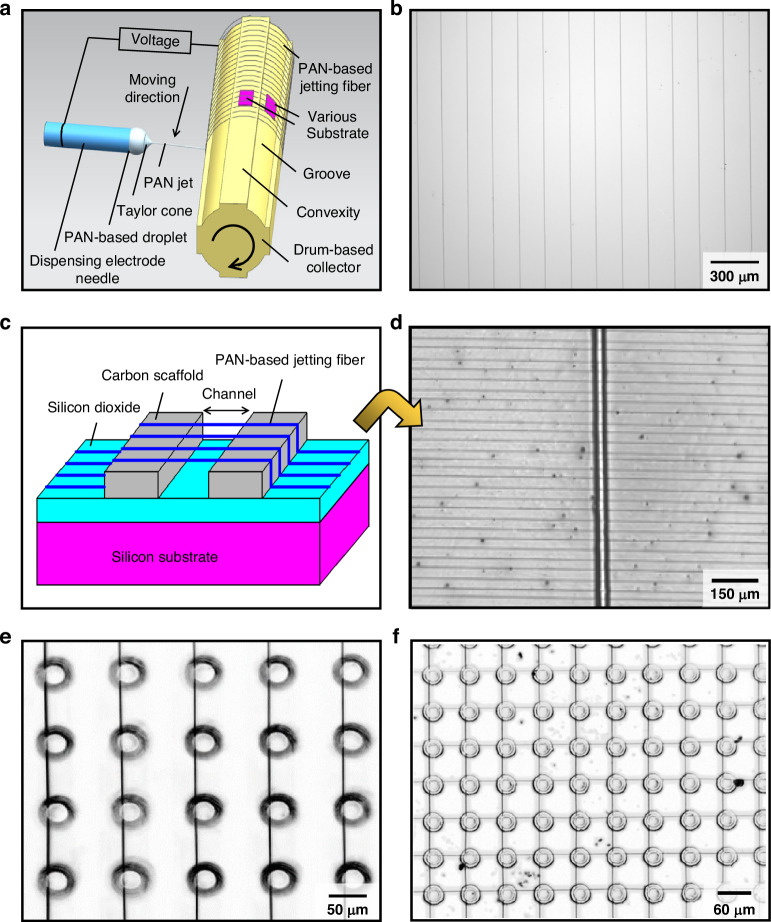
. **Additive of PAN jetting fiber with core-shell structure for micro/nano-structures** **a** Schematic additive of PAN jetting fiber to various substrates by a rotating drum. **b**–**e** Arrays of PAN jetting fiber on the surface of a silicon substrate in **b** as well as carbon micro-scaffolds in **c**, **d** and carbon micro-pillars in **e**. **f** Patterns of micro- and nanostructures consisting of PAN jetting fibers and carbon micro-pillars

Using the nano-structuring process and the submicro/nano-forming methods (supplementary materials) as described in Figs. 2–3 and Fig. S1–S3, a PAN jet on the meniscus of PAN-based droplet is formed in the range from submicron to nanometer by virtue of developing a Taylor cone, as shown in Fig. 4a. The resulting fibers are continuously deposited onto various substrates by moving the dispensing electrode needle along the surface of the drum-based collector in the guidance of the moving direction in Fig. 4a, enabling additive of PAN jetting fiber.

With the silicon substrate being selected and mounted onto the drum-based collector, an array of PAN jetting fiber is obtained in Fig. 4b, showing the uniform distance determined by the rotational speed of drum-based collector and the moving speed of the dispensing electrode needle^18^. Upon having carbon micro-scaffolds or carbon micro-pillars prepared on silicon substrates by the use of the C-MEMS method^23^, arrays of PAN-based jet fiber are able to be singly additively materialized on the channel between carbon scaffolds as well as the surface of carbon micro-scaffolds and silicon dioxide, as shown in Fig. 4c–e. Moreover, patterns of PAN jetting fiber on the surface of carbon micro-pillars in Fig. 4f are prepared by the secondary use of the additive. With the control of those fiber-forming voltages from 500 V to 0 V in the process of additive, the diameter of PAN jetting fiber can be tuned in the submicron to nanometer range, enabling the additive of submicro/nano-fiber on a silicon substrate or on the surface of silicon dioxide, micro-scaffolds, and micro-pillars as well as the channel between micro-scaffolds. These indicate that the additive nanostructuring based on the submicro-to-nano forming and nano-structuring process is capable of materializing core-shell submicron/nano-fibers to multifarious wafers for the fabrication of nano-structures on semiconductor devices.

**Fig. 5:**
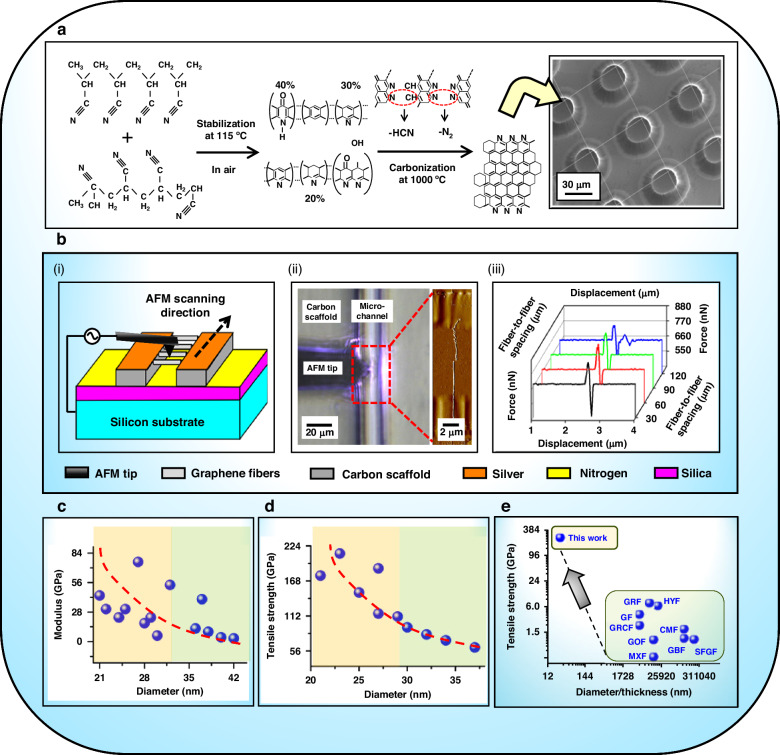
**Continuous carbon fiber with ultra-high strength by carbonization of PAN jetting fiber with core-shell structure** **a** Reaction path from continuous PAN jetting fiber to carbon fiber. **b** Schematic measurement (**i**), AFM images (**ii**), and force-displacement curves (**iii**) of carbon fiber suspended between carbon scaffolds. **c**, **d** Modulus (**c**) and tensile strength (**d**) of carbon fiber by varying the diameter. **e** Tensile strength of various fiber including of cellulose-based macrofibres (CMF)^24^, graphene-based fiber (GBF)^25^, graphene fiber (GF)^26^, graphene oxide-based fiber (GOF)^27^, graphene reinforced carbon fiber (GRCF)^28^, graphitic fiber (GRF)^29^, hybrid fiber (HYF)^30^, MXene fiber (MXF)^31^, silk fibroin reinforced graphene fiber (SFGF)^32^

The core-shell submicron/nano-PAN-fibers with these multifarious wafers in Fig. 4b–f are stabilized at 115 °C in air and then carbonized into continuous carbon fibers at 1000 °C in a nitrogen atmosphere, as shown in Fig. 5a. Following schematic measurement in Fig. 5b–i, atomic force microscopy (AFM) is used to stretch the carbon fibers in an array that is suspended between the carbon scaffolds until the breakage of the fibers occurs at the middle of the microchannel between the carbon scaffolds in Fig. 5b-ii. Force-displacement curves along AFM scanning direction in Fig. 5b-iii are accompanied by the breakage of the fibers. The forces (*F*_*a*_) and displacement (Δ*x*) in the force-displacement curves as well as the sizes of carbon fiber in the AFM images are substituted into *σ*_*ɸ*_ = *F*_*σ*_[(*d*_*a*_/2*Δx*)^2^ + 1]^1/2^/*πr*^2^, and *E*_*ɸ* _= *F*_*a*_*d*_*a*_/{2π*r*^2^ [(*d*_*a*_/2*Δx*)^2^ + 1]^1/2^ [(*d*_*a*_/2)^2^ + (*Δx*)^2^-*d*_*a*_] ^1/2^} where *F*_*σ*_ is the maximum force in the force-displacement curve, *σ*_*ɸ*_, *E*_*ɸ*_, *d*_*a*_, and *r* are the tensile strength, modulus, length, and diameter of carbon fiber, respectively. Using the results from the calculations of *σ*_*ɸ*_ and *E*_*ɸ*_, the decreasing trends of modulus and tensile strength with respect to the diameter in Fig. 5c, d are revealed for carbon fibers, elucidating the influence of scaling effects.

Moreover, the tensile strength of this carbon fiber up to 212 GPa in Fig. 5d far exceeds that of existing mainstream fibers including of CMF, GBF, GF, GOF, GRCF, GRF, HYF, MXF, SFGF (Fig. 5e), thus emphasizing superior mechanical properties. The nanoscale for the carbon fibers demonstrates the possibility of improving the tensile strength to far exceed that of those existing carbonaceous fibers^24–32^ by highlighting the nanoscale effect. Furthermore, the enhancing effect of the zigzag conformation formed in PAN fibers on the mechanical properties of carbon fibers^11^ further allows the tensile strength to be strengthened to ultra-high levels by the combined effects of the carbon-fiber-precursor microstructure and nanoscale, as shown in this paper. In addition, this zigzag conformation is not limited to enhancing mechanical properties, but more importantly contributes to further deriving multiple functions of carbon fibers, such as superhydrophilicity, piezoelectric properties, and cycling stability^11^. This nano-technology of ultra-strong carbon nanofiber derived from the zigzag conformation and the carbon-fiber-based nano-forming in this paper creates an opportunity for ultra-multifunctionality related to carbon nanofibers.

## Conclusion

In this work, we emphasize that the method of additive nanostructuring and carbonization is developed by the nanoforming method, the nano-structuring process, and subsequent carbonization for PAN jetting fiber. The nano-forming method of PAN jetting fiber is clearly elaborated by establishing mathematical models, designing the nano-forming processes and analyzing those results from characterization. Using this method in combination with shear stress theory, the nano-structuring process shows the ability to modulate the conformations of molecular chains, achieving the zigzag configuration inside the PAN jetting fiber. The zigzag conformations underlines the ability to highlight the unexpectedly high piezoelectric properties^12,33^, and the applications in the advanced self-charging supercapacitor^11^. Moreover, the zigzag configuration can be used to form the core-shell microstructure consisting of a zigzag configuration on the outside and helical configuration on the inside.

Using the method of additive nanostructuring and carbonization, the core-shell PAN nanofiber with the average diameter of ~80 nm can be fabricated and be additively materialized on the various substrate or micro-structures in the form of arrays on the various micro-structures or macro-substrate. The nano-size of such PAN fibers offers opportunities for super-performances of carbon fibers developed by pyrolyzing PAN fiber, such as high current density^18^, ultrastable current^18^, and ultra-low electron transfer rate^34^. The combination of nano-size and core-shell structure for such PAN fiber in this work offers further perspective on enhancing the effect of scale and structure on the properties in potential applications, such as absorption-dominated electromagnetic interference shields^35^, electrocatalytic performance enhancers^36^, high-rate supercapacitors^37^, and capacitive deionization devices^38^. In anticipation of even more prominent features, the additive nanostructuring and carbonization of PAN jetting enables the continuous carbon fiber to have a tensile strength of 212 GPa that far exceeds the strength of graphene-based fibers (724 MPa)^25^, graphitic fibers (6.57 GPa)^29^, silk fibroin reinforced graphene fibers (938.6 MPa)^32^, hybrid fibers (6.05 GPa)^30^, graphene oxide-based fibers (935 MPa)^27^, sustainable high-strength macrofibres (1.90 ± 0.32 GPa)^24^.

## Supplementary information

Supplementary information
