# Supplementary material for: Continuously superior-strong carbon nanofibers by additive nanostructuring and carbonization of polyacrylonitrile jetting
Source: Microsyst Nanoeng. 2024 Dec 10;10:185. doi: 10.1038/s41378-024-00800-7 (PMC11631983; doi:10.1038/s41378-024-00800-7)
Supplement: Supplementary file 1 — Supplementary information [file 41378_2024_800_MOESM1_ESM.docx]

Supplementary Document

**Jufeng Deng^1^, Chong Liu^2^, Marc Madou^3,4^**

^1^Key Laboratory of Advanced Manufacturing Technology of the Ministry of Education, Guizhou University, China, 550025

^2^School of Mechanical Engineering, Dalian University of Technology, China, 116023

^3^Mechanical and Aerospace Engineering, University of California, Irvine, USA, 92617

^4^School of Engineering and Science, Tecnologico de Monterrey, Mexico, 64849

**Methods**

**Submicron-forming of PAN jetting fiber**

From the preceding theories for the nano-forming of PAN jetting fiber, the fluid was initially pretreated by oxidation with the control of temperature at 106 ºC in accordance with previous report^17^, obtaining the shear-thinning property. The shear-thinning enables a reduction for the viscosity of fluid in jetting. Furthermore, a contact mode is introduced by decreasing the axial distance between the fluid and the collector and bringing the droplets into contact with the drum for the decrease in the fluid width at the tip of the dispensing electrode needle. In these conditions, the electrical stress at the lower threshold voltage in Eq. 5 is maximized in jetting due to a smaller liquid viscosity, a lower fluid-collector distance and a smaller fluid width. A very high local electric field for the jetting initiation and the formation of PAN jetting fiber is accompanied by the maximation of the electrical stress. Summarizing the above operations and analysis, the process for forming PAN jetting fiber is designed as described in Fig. S1-a.


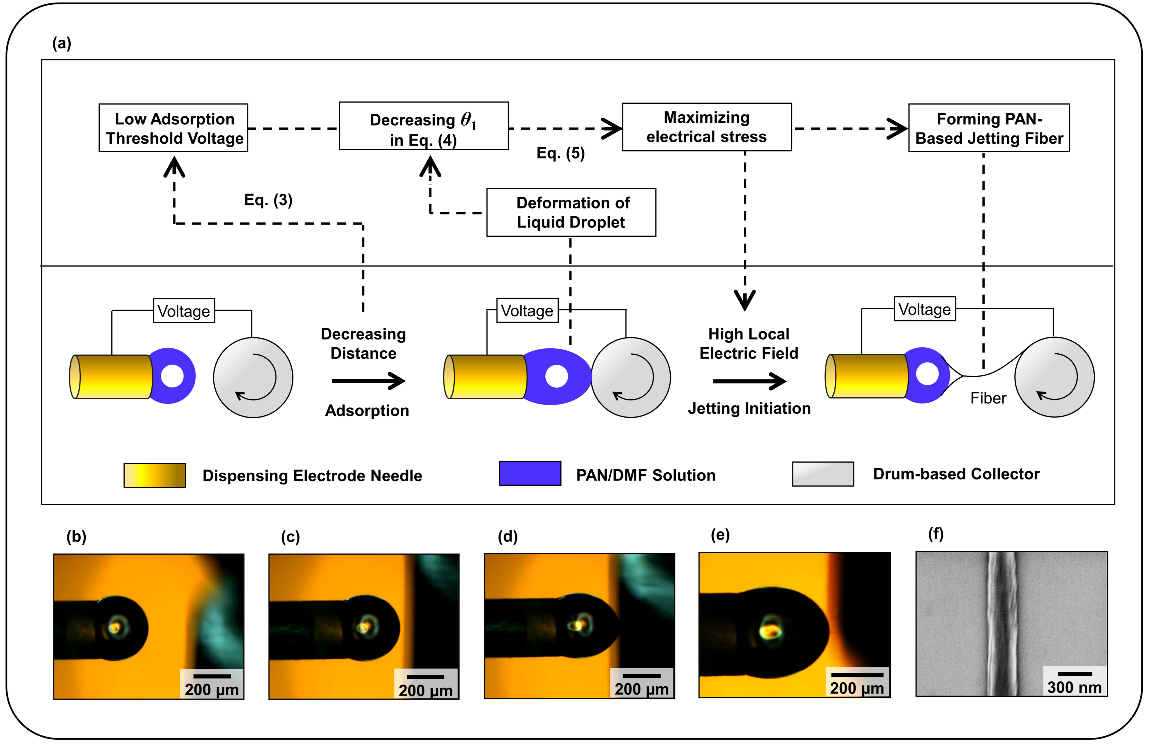


**Figure S1 Submicron-forming of PAN jetting fiber based on electrostatic jetting initiation at 500 V.** **a** Schematic design of the submicron-forming process for PAN jetting fibers. **b**-**e** Evolution from a droplet to a PAN jetting fiber by decreasing the spacing between the dispensing electrode needle and the drum-based collector in **(b)** and **(c)**, contacting from the electrostatic droplet to the drum-based collector in **(d)**, initiating a continuous jet and forming PAN jetting fibers in **(e)**. **f** Scanning electron microscopy image of a submicron -fiber derived from a PAN-based jet in **(e)**.

By the use of this process design, the spacing between the dispensing electrode needle and the drum-based collector is reduced at an applied voltage of 500 V by moving the dispensing electrode needle, as shown in Figure S1-b-d. As the reduction for needle-drum distance makes the droplet charged at the tip of the needle come closer to the drum, the electrostatic stress (Eq. 5) continues to increase and then makes the adsorption of the liquid droplet onto the collector surface in Figure S1-d. The adsorption of the droplet changes its shape from being circular to being elliptical, reducing the fluid width the tip of the dispensing electrode needle. The electrostatic stress can be maximized by the further decrease in the fluid width, initiating the jetting as shown in Figure S1-e. The formation of PAN-based fiber with a diameter of ~260 nm in Figure S1-f is accompanied by the jetting initiation. These results indicate that the process does enable the design based on the theoretical analysis of the electric stress to achieve the submicron-forming of PAN jetting fibers.


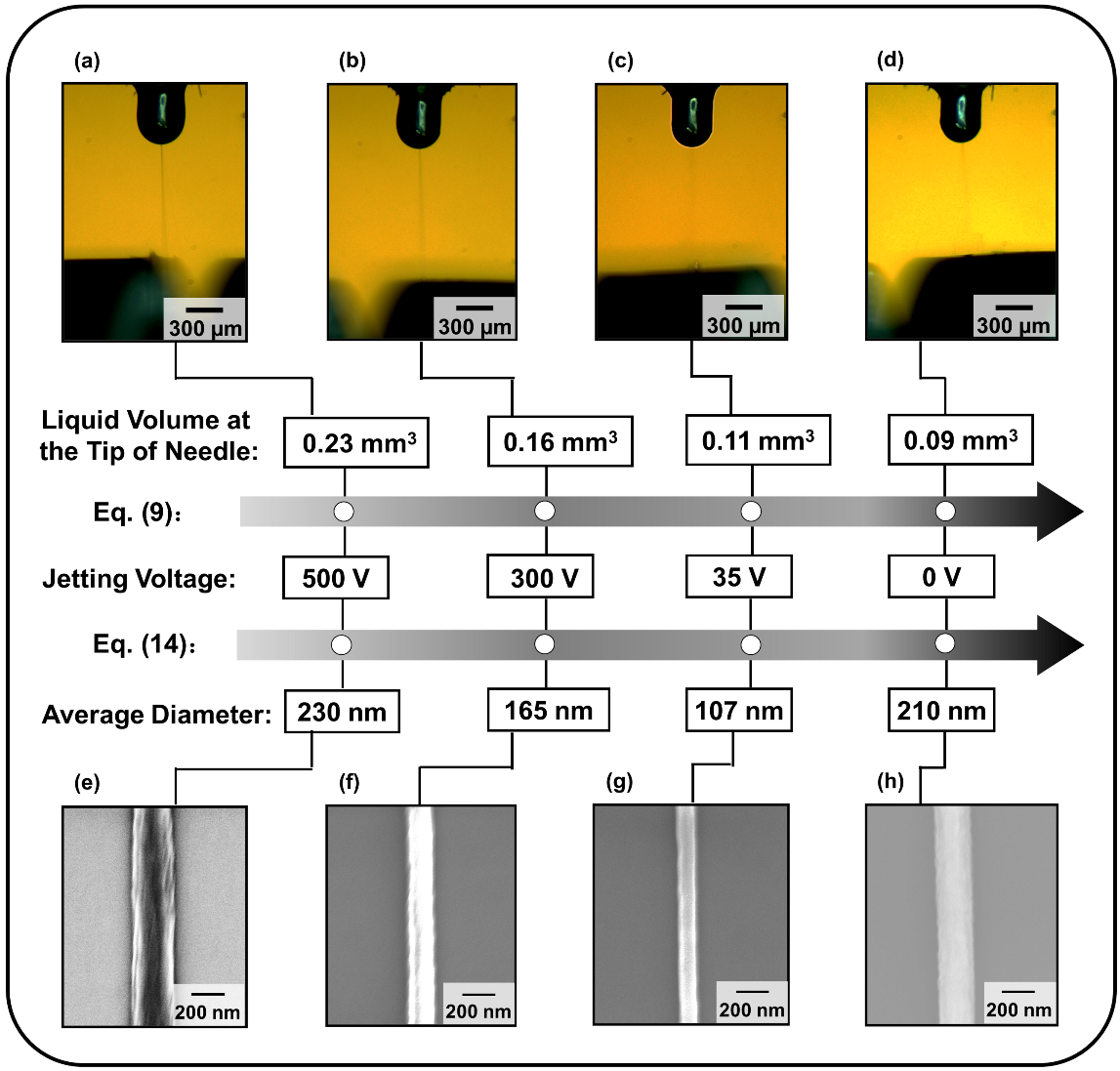


**Figure S2 Transition from submicron to near-nanometer in the diameter of PAN jetting fibers. a-d** Reduction of the liquid volume at the tip of the needle from 0.23 mm^3^ **(a)** to 0.09 mm^3^ **(d)**. **e-h** Scanning electron microscopy images of PAN fibers derived from these jets in **(a)-(d)**, respectively.

**Submicron-to-nanoscale forming of** **PAN jetting fiber**

In the case of the change from submicron to nanoscale for the forming of PAN jetting fiber, the steady-state voltage is minimized for the decrease in the diameter of PAN jetting fiber based on the analysis of Eq. 14. Among the factors affecting the minimum steady state voltage in Eq. 9, the liquid area at the nozzle exit A_N_ is chosen. By reducing the flow rate of the liquid, the liquid volume at the nozzle exit as the key parameter controlling A_N_ is decreased from 0.23 mm^3^ to 0.09 mm^3^. The decrease of the liquid volume can change the minimum steady-state voltage from 500 V to 0 V (see Figure S2-a-d). The resulting jet fibers at various voltages were characterized by scanning electron microscopy (SEM), showing a variation in average diameter from 230 nm to 210 nm. In the range of voltage from 500 V to 35 V, the average diameter versus the minimum steady-state voltage decreases as expected from Eq 14 for the case for *V*_min_ = constant. In contrast, the increase of the fiber diameter with decrease of voltage from 35 V to 0 V is also consistent with Eq 14 for the case of *V*_min_ → 0. These results show that the control of liquid area at the tip of the needle enables a near-nanoscale resolution of 195 nm for the forming of PAN jetting fibers at 35V.


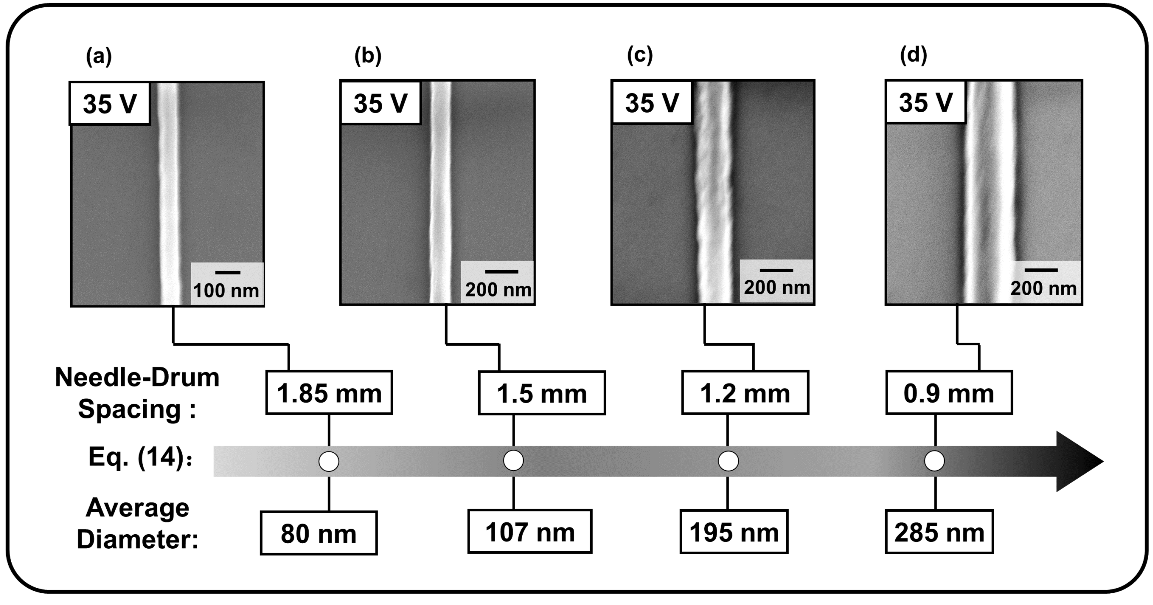


**Figure S3 Nano-formation of PAN jetting fiber. a-d** SEM images of PAN jetting fiber at the minimum steady-state voltage of 35 V with these needle-drum spacings of 1.85 mm **(a)**, 1.5 mm **(b)**, 1.2 mm **(c)** and 0.9 mm **(d)**, respectively.

Based on the effect of needle-drum distance on the diameter of PAN jetting fiber in Eq. 14, the resolution for the forming of PAN jetting fibers at 35 V can be further improved by increasing in the needle-drum distance. As the needle-drum distance increases from 0.9mm to 1.85mm in Figure S3, the resulting fibers exhibit a decrease in average diameter from 285 nm to 80 nm. This indicates that the nano-forming process consisting of the submicro-forming process and the controls of minimum steady-state voltage and needle-drum distance can produce PAN-based nanofiber in average diameter of 80 nm.
